# Prickle1‐driven basement membrane deposition of the iPSC‐derived embryoid bodies is separable from the establishment of apicobasal polarity

**DOI:** 10.1111/cpr.13595

**Published:** 2024-01-07

**Authors:** Dianlei Guo, Sikai Liu, Jiao Zhang, Xinyu Gu, Lei Shi, Yingchun Su, Shujuan Xu, Rong Ju, Yanhong Wei, Chunqiao Liu

**Affiliations:** ^1^ State Key Laboratory of Ophthalmology, Zhongshan Ophthalmic Center Sun Yat‐sen University Guangzhou China; ^2^ Department of Toxicology, School of Public Health Sun Yat‐sen University Guangzhou China; ^3^ Guangdong Provincial Key Laboratory of Brain Function and Disease Guangzhou China

## Abstract

Basement membrane (BM) component deposition is closely linked to the establishment of cell polarity. Previously, we showed that Prickle1 is crucial for BM deposition and cell polarity events in tear duct elongation. To gain a deeper understanding of the intimate relationship between BM formation and cell polarity, we generated induced pluripotent stem cells (iPSCs)‐derived embryoid bodies (EBs) with a basement membrane separating the visceral endoderm (VE) and inner EB cell mass. We found that *Prickle1* was highly expressed in VE of the normal EBs, and the *Prickle1* mutant EBs displayed severely impaired BM. Notably, the formation of the basement membrane appeared to rely on the proper microtubule network of the VE cells, which was disrupted in the *Prickle1* mutant EBs. Moreover, disruption of vesicle trafficking in the VE hindered BM secretion. Furthermore, reintroducing Prickle1 in the mutant EBs completely rescued BM formation but not the apicobasal cell polarity of the VE. Our data, in conjunction with studies by others, highlight the conserved role of Prickle1 in directing the secretion of BM components of the VE cells during embryonic germ layer differentiation, even in the absence of established general polarity machinery. Our study introduces a novel system based on iPSCs‐derived EBs for investigating cellular and molecular events associated with cell polarity.

## Graphical abstract

In this work, Guo et al. described the differentiation of embryoid bodies (EBs) from iPSCs derived from control and the *Prickle1* mutant mice and utilized this system to dissect the connections between basement membrane (BM) deposition and apicobasal (AB) polarity. Illustrated are differentiated embryoid bodies with integral BM and AB polarity in the control EBs, disrupted BM and AB in the Prickle1 mutant EBs, and restored BM but not AB polarity in the ‘Prickle1‐rescue’ EBs. These results suggest that Prickle1 dictates BM deposition separable from its function in AB polarity, which is not as conventionally thought. Meanwhile, the study highlights the importance of hypoblast‐derived visceral endoderm (VE) in BM deposition of the early embryonic germ layers.

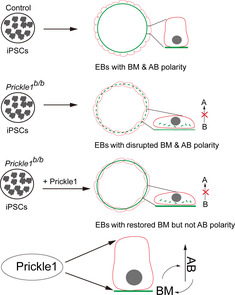

## 1 INTRODUCTION

Basement membrane (BM) exists in nearly all tissues, forming from the supramolecular assembly of extracellular matrices (ECM) at the base of epithelial cells. BM primarily consists of type IV collagen (Col IV), Laminin, Nidogen, and heparan sulfate proteoglycans such as Perlecan or Agrin.^1^ Mutations in BM components lead to a variety of systemic diseases, including Alport's, Pierson, and Schwartz‐Jampel syndromes.^2^, ^3^ BM provides tissue barriers and mechanical support for morphogenesis and functions.^1^, ^4^ It also serves as a signalling platform to coordinate neighbouring cell behaviours such as polarization and migration.^5^, ^6^, ^7^, ^8^, ^9^, ^10^

Studies in several species and in vitro systems suggested that BM is essential for establishing cell and tissue polarity. For example, in Drosophila, Dystroglycan, a receptor for Laminin and Perlecan and a transmembrane linkage between extracellular BM and cytoskeletons, is crucial for cell apicobasal and anterior–posterior polarity of the follicular epithelium.^9^, ^11^ In MDCK cells, basal laminin assembly mediated by Rac1 is required for the maintenance of the lumen apicobasal polarity in cyst formation.^12^ Additionally, in ascidian notochord development, *chongmague* (*chm*), an ortholog of vertebrate alpha 3/4/5 family of Laminin genes, plays an important role in midline convergent extension and maintenance of tissue boundaries, which involve planar cell polarity (PCP) signalling.^10^ Laminin polymerization also induces receptor‐cytoskeleton intracellular reorganization, suggesting the cytoskeleton's role in the crosstalk between BM and cell polarity.^13^

BM proteins are synthesized and deposited to the basal extracellular space of the cells through designated intracellular routes. In Drosophila, Col IV and Laminin mRNAs and proteins are localized to the basal ER compartment.^6^ RNAi analysis demonstrated that BM proteins are exported to the basal Golgi clusters through Tango1‐positive ER exit sites with coordination of Crag and Rab10.^6^ Crag is localized to the apicolateral domain of Drosophila follicular epithelium, controlled by phosphatidylinositol 4,5‐bisphosphate (PIP2) level to prevent apical transport of the BM basal secretion.^14^ Both Tango1 and Rab10 are planar polarized at the base of the follicular epithelium, whereas Crag, broadly distributed along the apicobasal axis, is also partially planar polarized at the base.^6^ Recently, Rab6 was also shown to be involved in transporting secretory cargos, including Laminin.^15^ Interestingly, in compromised BM secretion due to Rab10 or phosphatidylinositol disruption, general cell polarity was essentially maintained.^6^, ^14^ Thus, it remains debated whether a causal‐consequential relationship between BM deposition and the establishment of cell polarity exists and, if so, how the two events are connected.

Epithelial cells are polarized along the apicobasal axis and tissue plane, attributing to distinct yet closely related sets of protein modules. The apicobasal polarity (AB) was characterized by apical localization of Crumbs/Pasl1/Patj and Par3/Par6/aPkc^16^ modules, while the planar polarity (PCP) utilizes a set of six proteins including Frizzled, Dishevelled, Vangl, Prickle, Diego and Flamingo to establish polarity.^17^, ^18^, ^19^, ^20^, ^21^ Whilst little is known about the connection between the classic AB modules and BM deposition, the PCP component, Vangl2, is crucial for BM integrity and localization of the mouse corneal epithelium.^22^ Furthermore, the ascidian *aimless* (*aim*), encoding an ortholog protein of the vertebrate PCP component, Prickle, is reported to regulate Laminin protein localization during embryonic axis development.^10^ Our recent study showed that disruption of *Prickle1* completely abrogates BM formation of the tear duct epithelium, with altered cell–cell adhesion and cell axis orientation to some degrees.^23^ Interestingly, Prickle1 is also reported to be involved in the epiblast AB polarity during mouse embryogenesis with compromised ECM deposition.^24^ Thus, a deep understanding of the role of Prickle1 in BM deposition will be the key to further elucidating the relationship between BM deposition and the AB or PCP cell polarity.

Before gastrulation, the mouse embryo consists of two germ layers, the epiblast and the visceral endoderm (VE), separated by the basal lamina/basement membrane (BM). While Prickle1 is essential for epiblast AB polarity during early embryogenesis, it is also expressed in VE by in situ hybridization.^24^ The role of Prickle1 in VE polarity and the potential contribution of VE to the epiblast defects in the *Prickle1* mutant embryos have yet to be addressed. The in vitro differentiated embryoid body (EB) from embryonic stem cells (ESCs) possesses well‐formed BM separating the inner EB cell mass from the polarized VE,^25^, ^26^ which serves as an ideal model to study Prickle1 function in VE polarity and BM deposition. Taking the advantages of EBs from the wild type and *Prickle1* mutant iPSCs (induced pluripotent stem cells), we investigated the molecular connections between the BM deposition and VE cell AB polarity. Our results revealed that *Prickle1* determines the proper BM basal secretion of the VE in the absence of general AB polarity machinery likely contributing to the epiblast polarity of the early embryos.

## 2 RESULTS

### 2.1 Generation and characterization of iPSCs from the MEFs

**FIGURE 1:**
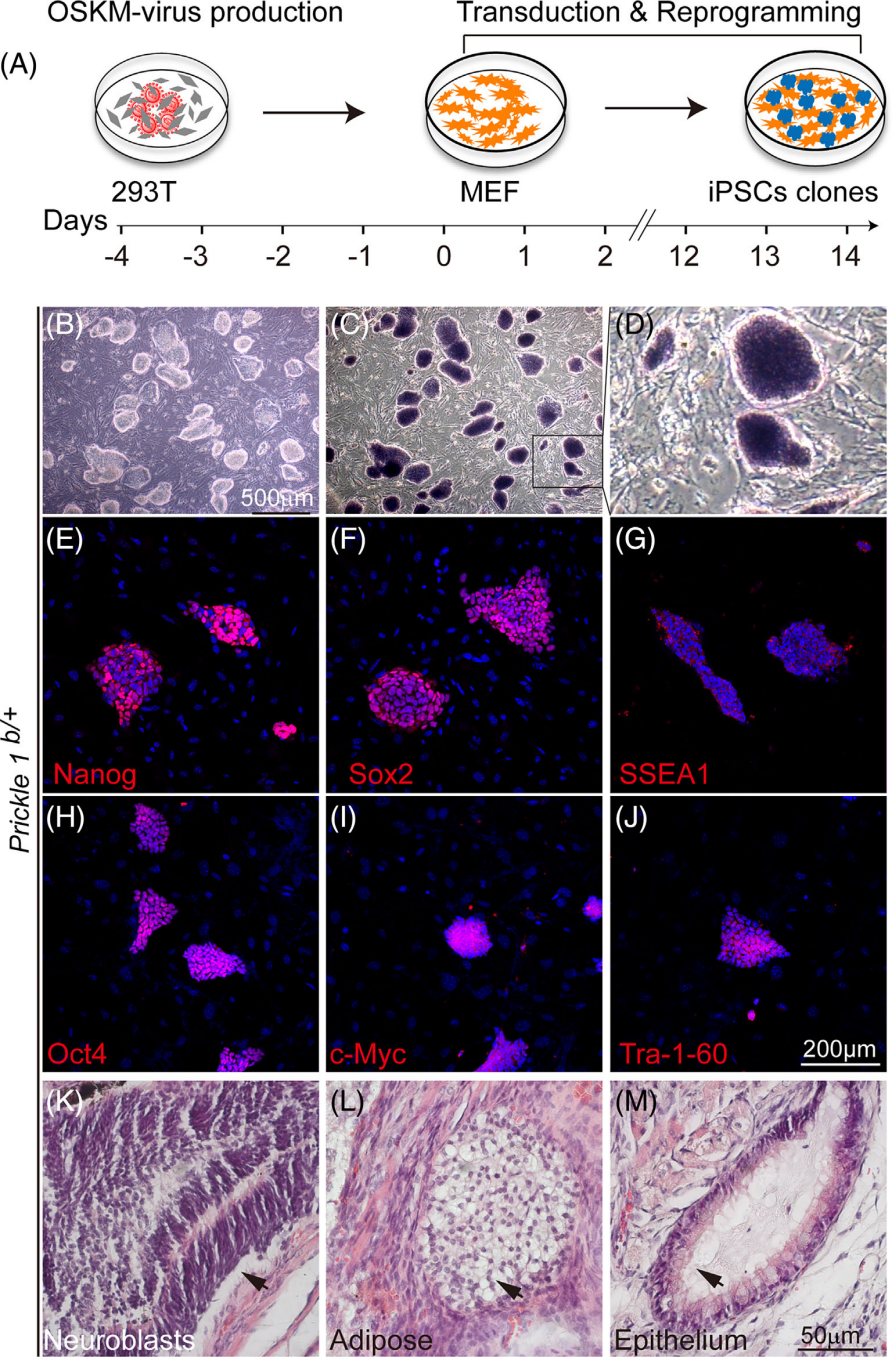
Generation and validation of iPSCs reprogrammed from MEFs. (A) Schematic illustration of generating iPSCs from reprogramming MEFs. HEK293T was used to produce the lentivirus particles carrying OSKM four transcription factors. The viral particles were used to transduce and reprogram the cultured mouse embryonic fibroblasts (MEFs) into induced pluripotent stem cells (iPSCs). (B) The typical morphology of iPSCs grown on feeder MEFs at low magnification under bright field microscopy. (C) X‐phos staining (dark blue) showing endogenous alkaline phosphatase activity of the iPSC clones. (D) Magnified image from the boxed area in (C). (E–J) iPSC clones immunostained for Nanog (E), Sox2 (F), SSEA1 (G), Oct4 (H), c‐Myc(I), and Tra‐1‐60 (J). (K–M) HE histology of teratoma tissues formed by iPSC implantation. Arrows point to tissues from iPSC‐differentiated ectoderm (neuroblasts) (K), mesoderm (adipose) (L), and endoderm (epithelium) (M).

We generated *Prickle1* ^*b/+*^ and *Prickle1* ^*b/b*^ mice previously.^23^ With these mice, *Prickle1* ^*b/+*^ and *Prickle1* ^*b/b*^ MEFs were prepared from the E13.5 embryos along with the wild type (Materials and methods).^27^ iPSCs were acquired by reprogramming the MEFs using the lentiviral vectors carrying ‘OSKM’ four transcription factors (4‐factor formula)^28^ (Materials and methods, Figure 1A). The OSKM‐induced clones exhibited typical iPSC morphology for all three genotypes (Supplementary Figure S1A–I, Figure 1B) and were positive for alkaline phosphatase activity (Figure 1C,D). They expectedly expressed routine iPSC markers such as Nanog, Sox2, Ssea1, Oct4, c‐Myc, and Tra‐1‐60 (Figure 1E–J, Supplementary Figure S1J–O). qPCR analysis of key pluripotency markers including Nanog, Oct4, Fgf4, Esg1, and Sox2 demonstrated no statistical expression differences between wild type (*Prickle1* ^*+/+*^), *Prickle1* ^*b/+*^, and *Prickle1* ^*b/b*^ iPSCs (Supplementary Figure S1P). Additionally, by intraperitoneal implantation of *Prickle1* ^*b/+*^ iPSCs into immunodeficient mice, teratoma was formed normally with typical three germ layer‐derived neuroblasts, adipose, and epithelium (Figure 1K–M).

Two iPSC lines (ht‐iPSC‐1 and hm‐iPSC‐1, Supplementary Figure S1D,G) were further subjected to karyotyping analysis showing grossly normal numbers and morphologies of the chromosomal pairs (Supplementary Figure S2A,B). Additionally, all clones used for later experiments (Supplementary Figure S1D–I) showed no ‘OSKM’ expression, suggesting that the observed viral vector integration loci were silenced (Supplementary Figure S2C,D). These results indicate a successful induction of iPSCs with standard molecular and functional features.

### 2.2 Defective *Prickle1* mutant EBs differentiated from the generated iPSCs

**FIGURE 2:**
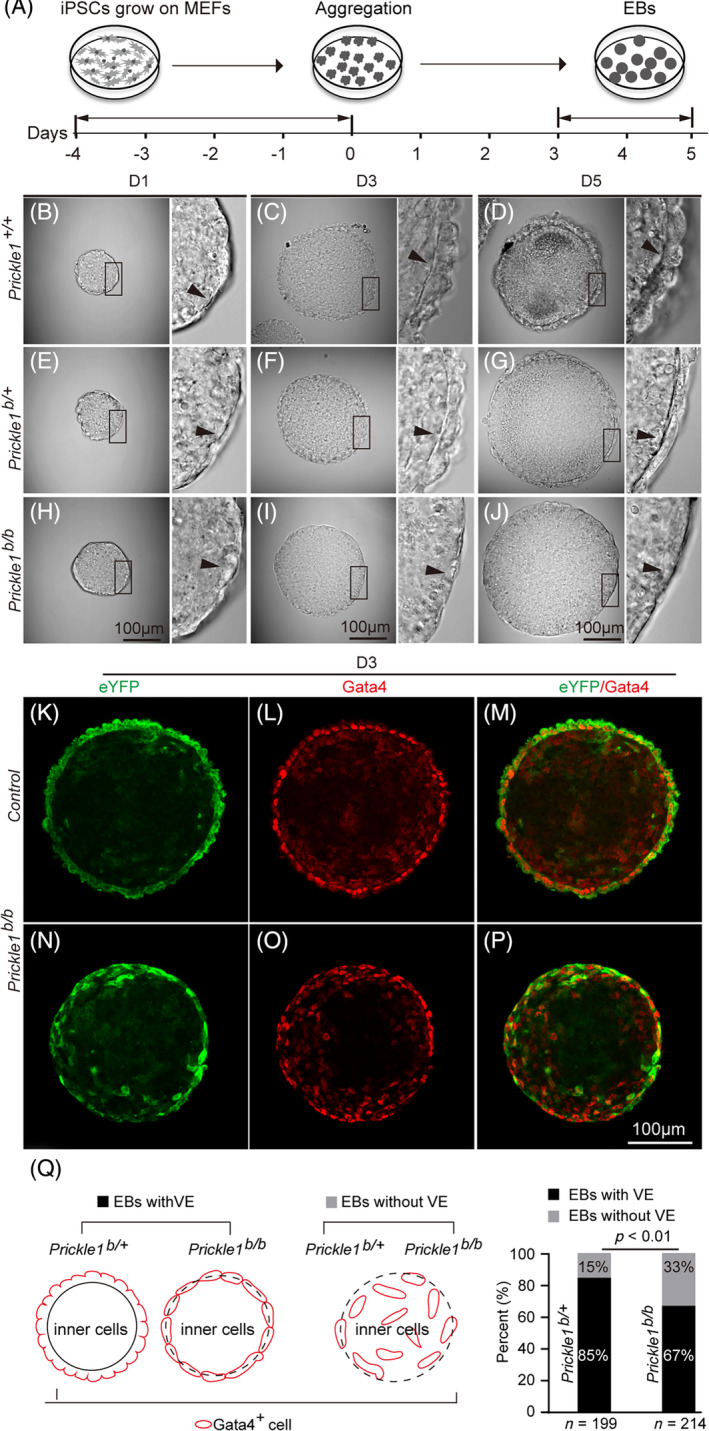
Differentiation of embryoid bodies (EBs) from the *Prickle1* ^*b/+*^ and *Prickle1* ^*b/b*^ iPSCs and identification of the visceral endodermal (VE) cells. (A) Schematic illustration of a workflow of EB differentiation. IPSCs were grown on MEFs for 4 days (D (−4)) followed by aggregation and cultured for another 5 days (D5). (B–J) Bright‐field microscopy of differentiated EBs at different time points. Boxed areas are magnified to the right. Arrowheads point to the presumptive VE layer. (B–D) Wild type EBs (*Prickle1* ^*+/+*^); (E–G), Control (*Prickle1* ^*b/+*^) EBs; (H–J) Mutant EBs (*Prickle1* ^*b/b*^). (K–P) Green: immunostaining for eYFP, a reporter knocked in the *Prickle1* locus; Red: immunostaining for Gata4, a marker for the VE cells. (K‐M) *Prickle1* ^*b/+*^ control EBs; (N–P) *Prickle1* ^*b/b*^ mutant EBs. (Q) Statistical analysis of successfully differentiated EBs indicated by Gata4 staining of the outer layer of VE (L, O). On the left is a schematic drawing of the EB with or without VE identified by Gata4 staining (red curved lines). EBs with differentiated VE were subjected for further experimental analysis. On the right are graphs of calculation of EBs with or without VE. A chi‐squared test was performed to detect statistical powers *p*‐values. Statistical significance was defined as *p* < 0.05.

We next test the capacity of iPSCs to differentiate into EBs. The acquired iPSCs were amplified and cultured for 4 days before reaching a desirable size of about 200 μm for EB aggregation and differentiation (Figure 2A). The typical and healthy‐looking EBs were mostly observed from day 3 (D3) to D5 of aggregation in both *Prickle1* ^b/+^ and the wild type (*Prickle1* ^+/+^) (Figure 2B–G). *Prickle1* ^+/+^ and *Prickle1* ^b/+^ EBs exhibited typical visceral endoderm and inner EB cell mass with a clear tissue boundary under the bright‐field light microscopy (Figure 2B–G). In contrast, the VE from the mutant EBs (*Prickle1* ^b/b^) were rather flattened, hardly distinguishable from the inner EB cell mass (Figure 2H–J, as arrows pointed), a sign of a defective VE.

Because *Prickle1* ^b/+^ EBs are structurally and morphologically normal as the wild type and carried an *eYfp* reporter at the endogenous *Prickle1* locus,^21^ which facilitates monitoring the spatiotemporal expression of *Prickle1*, we utilized them as controls for the rest of this study. We went on to identify the presumptive VE and inner EB cell mass of the differentiated EBs. The control and the mutant VE displayed high expression of the eYFP reporter indicated by an antibody against eYFP (also GFP) (Figure 2K,N,M,P, green). The VE fate was further indicated by co‐staining of Gata4 (Figure 2L,O,M,P, red), a transcription factor for VE, with eYFP in the outer layer of EBs (Figure 2K,N). Chi‐square statistical analysis showed that *Prickle1* ^b/b^ mutant EBs had a lowered differentiation efficiency in terms of Gata4 positive cells in the outer EB compared with the controls (67% vs. 85%, Figure 2Q). Furthermore, all inner EB cell mass of the controls and mutants highly expressed Oct4 (Supplementary Figure S3A,B) and Nanog (Supplementary Figure S3C,D), markers for embryonic inner cell mass tissue. Thus, the generated iPSCs were able to differentiate into EBs with typical structures expressing a set of distinct markers.

### 2.3 Prickle1 is required for BM basal deposition and columnar shape of the visceral endoderm (VE) cells

The observed defective VE was characterized by the flattened outer surface on the *Prickle1* ^b/b^ EBs (Figure 2H–J, N–P), a polarity event probably related to vectorial BM deposition during tear duct development.^23^, ^29^ To further investigate whether BM was also altered in the *Prickle1* mutant EBs, we examined the expression and localization of the principal components of the BM in the differentiated control and *Prickle1* ^*b/b*^ mutant EBs at D3.

**FIGURE 3:**
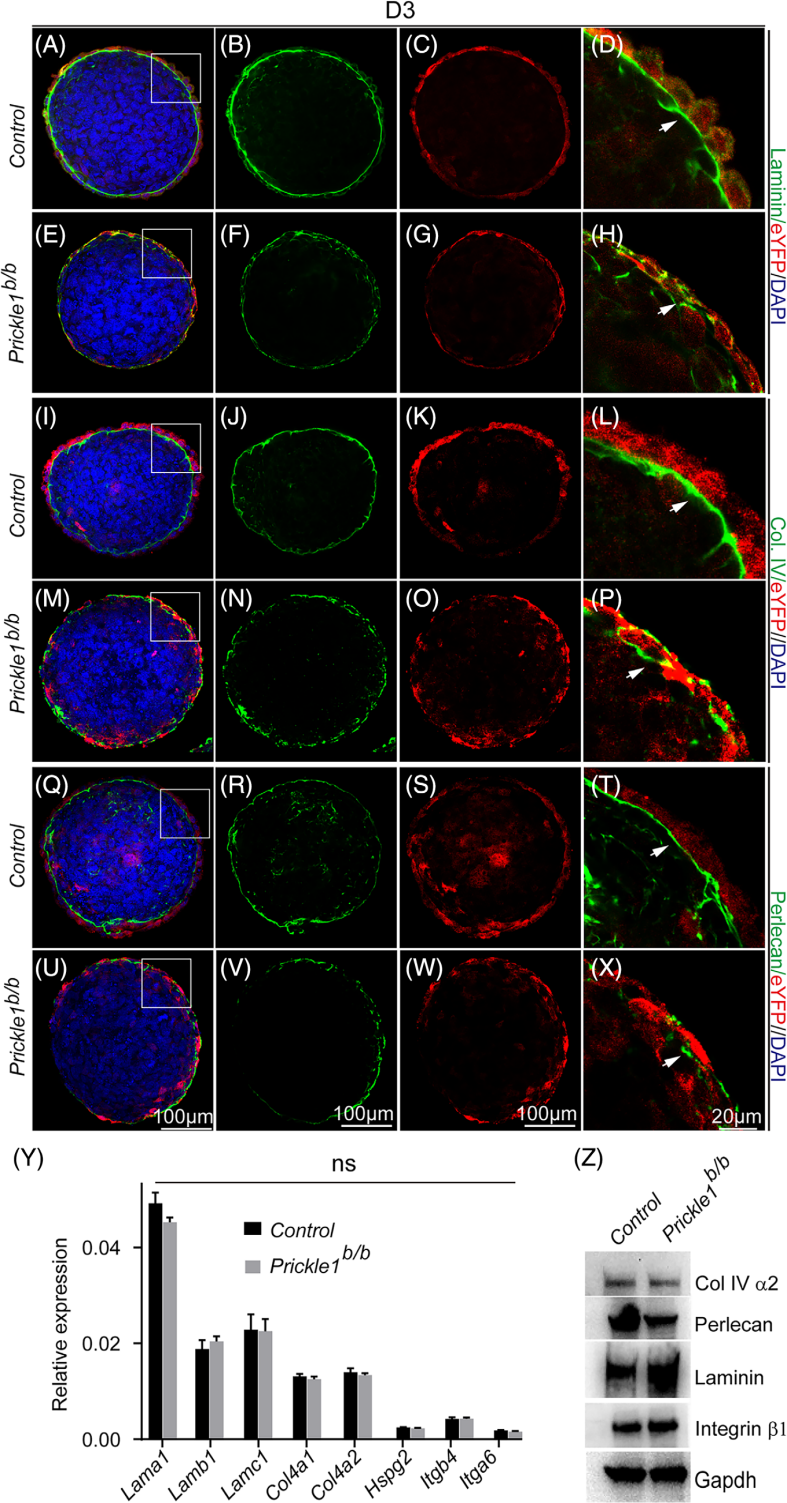
Detection of basement membrane (BM) components of Laminin, Col. IV, and Perlecan in the control and mutant EBs. (A–H) Immunohistochemistry to detect Laminin (green) and eYFP (red). DAPI stained nucleus. (A–D) Control EBs; (E–H) Mutant EBs. (I–P) Immunohistochemistry to detect Col. IV (green) and eYFP (red). DAPI stained nucleus. (I–L) Control EBs; (M–P) Mutant EBs. (Q–X) Immunohistochemistry to detect Perlecan (green) and eYFP (red). DAPI stained nucleus. (Q–T) Control EBs; (U–X) Mutant EBs. The leftmost column is merged images from their corresponding rows of separate channels. Boxed areas were magnified on the rightmost column with arrows pointing to the BM. (Y), RT‐qPCR determined the mRNA expression of genes encoding BM components. *Lama1*, *Lamb1*, and *Lamc1* for Laminin; *Col4a1* and *Col4a2* for Col. IV; *Hspg2* for Perlecan; *Itgb4* and *Itga6* for integrin. RT‐qPCR CT values were normalized to the *Gapdh*, serving as an internal control (Materials and Methods), and plotted as bar graphs. Student *t*‐test was performed to detect statistical powers‐ *p*‐values. Statistical significance was defined as *p* < 0.05. (Z), Representative images of western blotting of BM proteins and a Laminin receptor, integrin β1. Gapdh serves as an internal control.

Disrupted Laminin assembly was consistently observed (Figure 3E–H compared with 3A–D). Similarly, lamina deposition of Col. IV (Figure 3M–P, compared with I–L) and Perlecan (Figure 3U–X, compared with Q–T) was also abrogated. To determine whether the lack of BM of the mutant EBs was due to compromised expression of BM components, we first performed RT‐qPCR to measure the mRNA levels of BM components. No significant expression difference between the control and the mutant EBs was detected for *Lam* (*a1*, *b1*, and *c1* for Laminin), *Col4 (a1* & *a2* for Col IV), or *Hspg2* (for Perlecan) (Figure 3Y). The expression of Laminin receptor genes *Itgb4 and a6* are also similar (Figure 3Y). We then examined the expression levels of corresponding proteins. Consistent with the mRNAs, we found no essential differences between the control and the mutant EBs (Figure 3Z). Thus, Prickle1 is likely required for the BM deposition rather than the BM component expression.

### 2.4 Altered cytoskeletons of the *Prickle1* ^*b/b*^ mutant VE and the effect of cytoskeleton disruption on BM secretion

**FIGURE 4:**
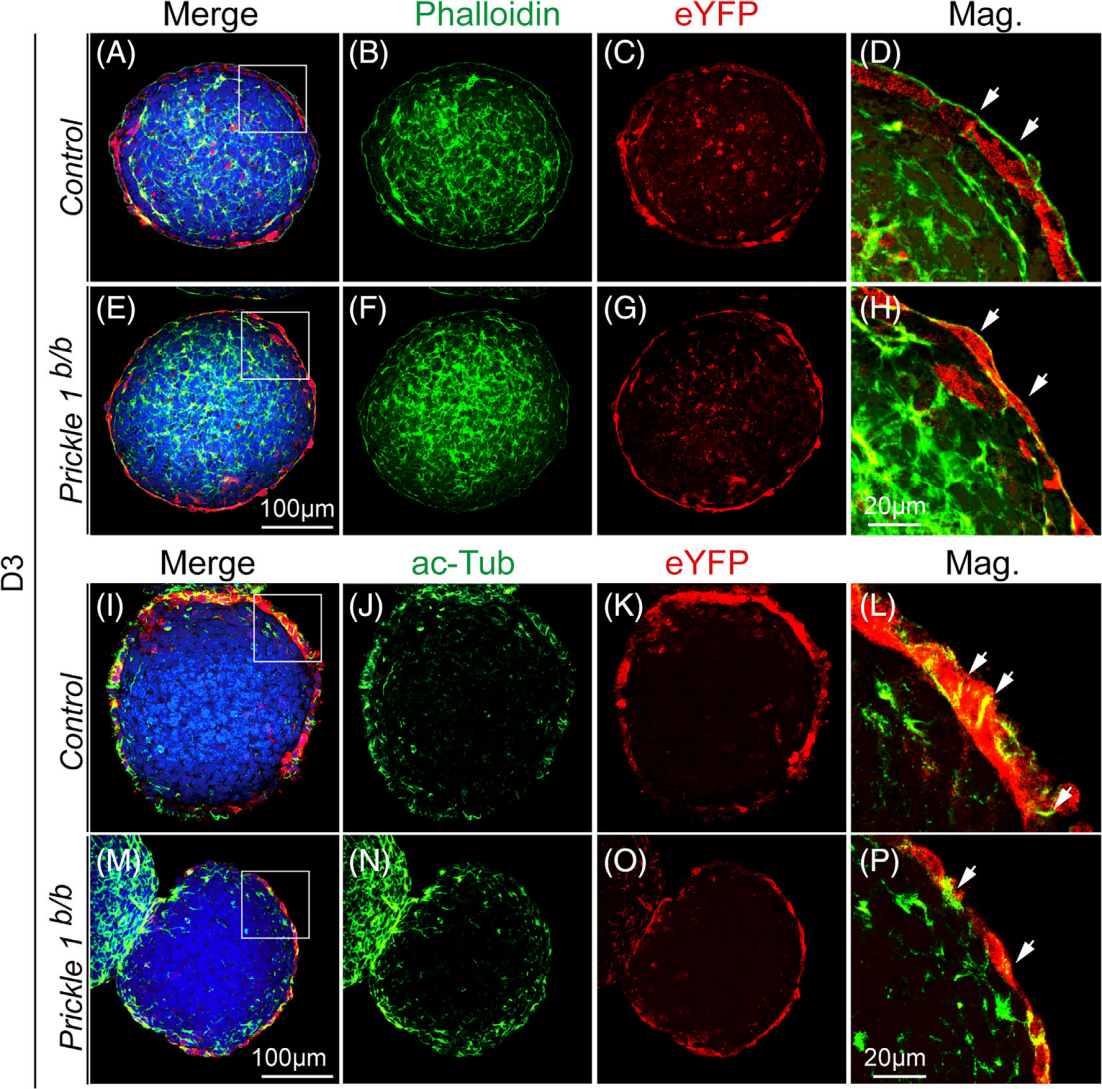
Altered cytoskeletons of the *Prickle1* ^*b/b*^ mutant VE. For all panels, boxed areas are magnified in the rightmost column. The leftmost column is merged images from their corresponding rows of separate channels. DAPI staining is in the blue channel for all panels. (A–H) Green, EBs stained with Phalloidin to detect actins; Red, EBs stained with an anti‐eYFP antibody to detect *Prickle1*‐expressing VE cells. Arrows point to the apical actin fibres. (A–D) Control EBs. (E–H) Mutant EBs. (I–P) Green, EBs stained with an ac‐Tub antibody to detect acetylated alpha tubulin; Red, EBs stained with an anti‐eYFP antibody to detect *Prickle1*‐expressing VE cells. Arrows in the control EBs point to the tubulin tracks, which were lost in the mutants. (I–L) Control EBs. (M–P) Mutant EBs.

The flattened VE cells of the *Prickle1* ^*b/b*^ mutant EBs indicated a cell polarity alteration closely associated with cytoskeletons, which may contribute to the polarized BM deposition. We, therefore, examined actin filaments and microtubule networks. In controls, while the actin fibres stained with phalloidin were found localized preferentially in the apical side of the VE cells (Figure 4A–D), a weaker but similar apical localization was detected in the *Prickle1* ^*b/b*^ mutant VE cells (Figure 4E–H). Furthermore, the microtubule tracks labelled with acetylated tubulin appeared considerably less in the mutant VE cells compared with the controls (Figure 4M–P compared with 4I–L).

**FIGURE 5:**
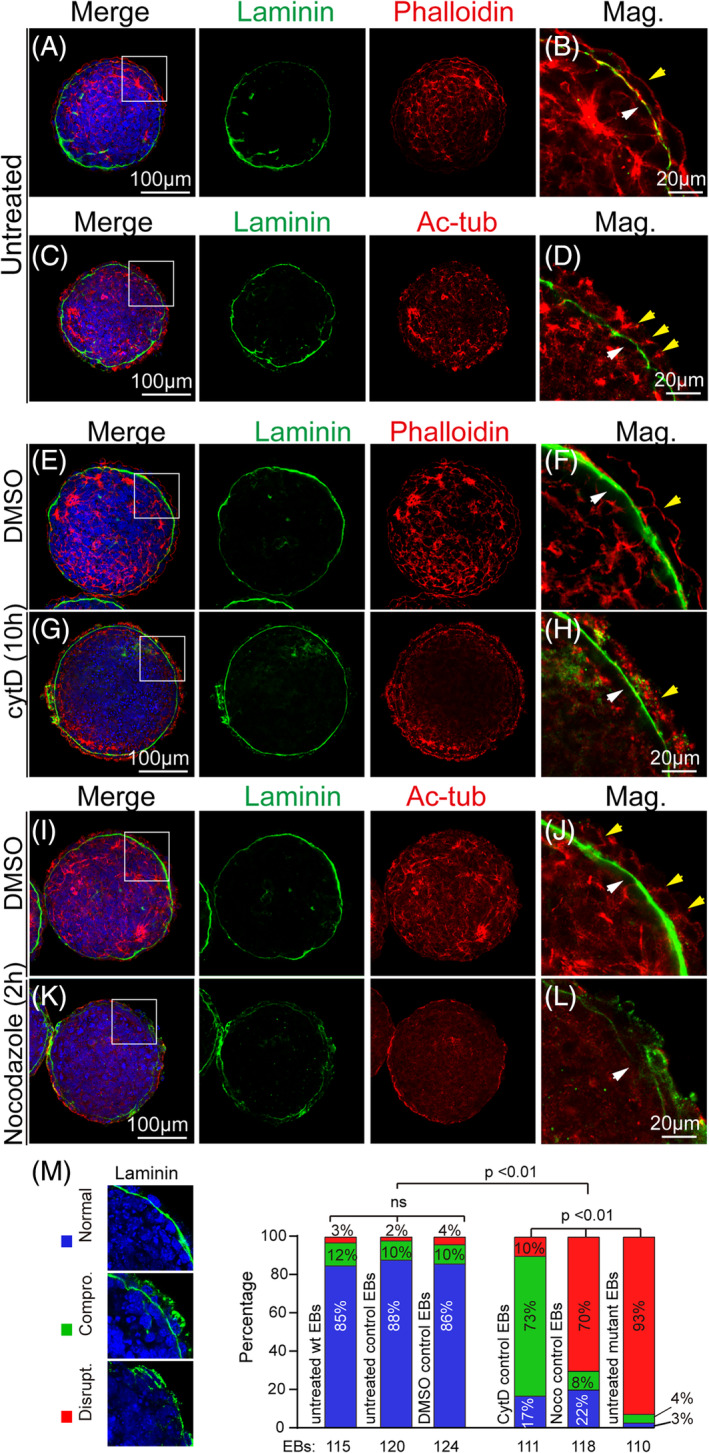
Effects of cytoskeleton disruption on BM formation. For all panels, boxed areas are magnified in the rightmost column. The leftmost column is merged images from their corresponding rows of separate channels. DAPI staining is in the blue channel for all panels. (A, B) Untreated control EB stained with Laminin (Green) and Phalloidin (Red). The white arrow points to the BM, and the yellow arrow points to the apical actin fibres. (C, D) Untreated control EB stained with Laminin (Green) and Ac‐tub (Red). The white arrow points to the BM, and the yellow arrows point to stained tubule tracks. (E–H) Green, EBs stained for Laminin; Red, EBs stained for Phalloidin; (E, F) DMSO‐treated control EBs. (F) The white arrow points to the BM, while the yellow arrow points to the actin fibres. (G, H) Cytochalasin D‐treated EBs for 10 hours (10 h). The yellow arrow points to the actin clumps in (H). (I–L) Green, EBs stained for Laminin; Red, EBs stained for ac‐tub; (I, J) DMSO‐treated control EBs; (K, L) Nocodazole treated EBs. The white arrow in (J) points to the BM, while the yellow arrows point to the tubule tracks. The white arrow in (L) indicates disrupted BM. (M) Quantification of EBs with the normal, compromised (Compro.), and disrupted (Disrupt.) BM upon drug treatments to disrupt cytoskeletons. The left column of images with Laminin staining (green) indicates the criteria to define normal, compromised, and disrupted EBs according to BM defective severity. Graphs in the right panel are percentages of different types of EBs. Blue bars represent normal EBs, while the green and red bars represent EBs with compromised and disrupted BM, respectively. A chi‐squared test was performed to detect statistical powers *p*‐values. Statistical significance was defined as *p* < 0.05.

Because the mutant EBs already had compromised cytoskeleton with no BM formation, which was not helpful to illustrate the cause‐and‐effect relationship between cytoskeleton alteration and the BM loss, we used drug inhibitors to disrupt cytoskeletons of the control EBs and observed the consequential BM changes. Untreated EBs showed clear BM and cytoskeleton staining patterns under and surrounding the VE, respectively (Figure 5A–D, arrows). Cytochalasin D‐treated EBs disrupted the actin fibres and compromised BM deposition as indicated by cytoplasmic Laminin retention till 10 h posttreatment (Figure 5E–H). In contrast, disruption of microtubule networks with nocodazole caused acute and severe BM disruption within a 2 h treatment window (Figure 5I–L). We further categorized the EBs into three groups according to the severity of the BM defects. EBs with integral BM and no cytoplasmic accumulation of laminin were designated as ‘normal’ (Figure 5M, blue icon); EBs with compromised BM structure and cytoplasmic laminin accumulation were designated as ‘compromised’ (Figure 5M, green icon); and EBs with on apparent BM structure were designated as ‘disrupted’ (Figure 5M, red icon). Chi‐square statistical analysis demonstrated that the DMSO‐treated group was similar to the untreated wild type or control group in terms of the formed number of EBs with normal BM deposition (Figure 5M). Consistently, the BM defective severity with cytoD treatment was less than the nocodazole treatment, and both were less severe than the untreated mutant EBs (Figure 5M). Further treatment with bafilomycin A1, an inhibitor for V‐ATPase proton pump of many types of trafficking vesicles, also led to the cytoplasmic trap of Laminin (Supplementary Figure S4). Taken together, the above results suggested that Prickle1 might affect Laminin vesicles trafficking preferentially in a microtubule‐dependent manner.

### 2.5 Reintroduction of Prickle1 into the mutant EBs restores BM formation

**FIGURE 6:**
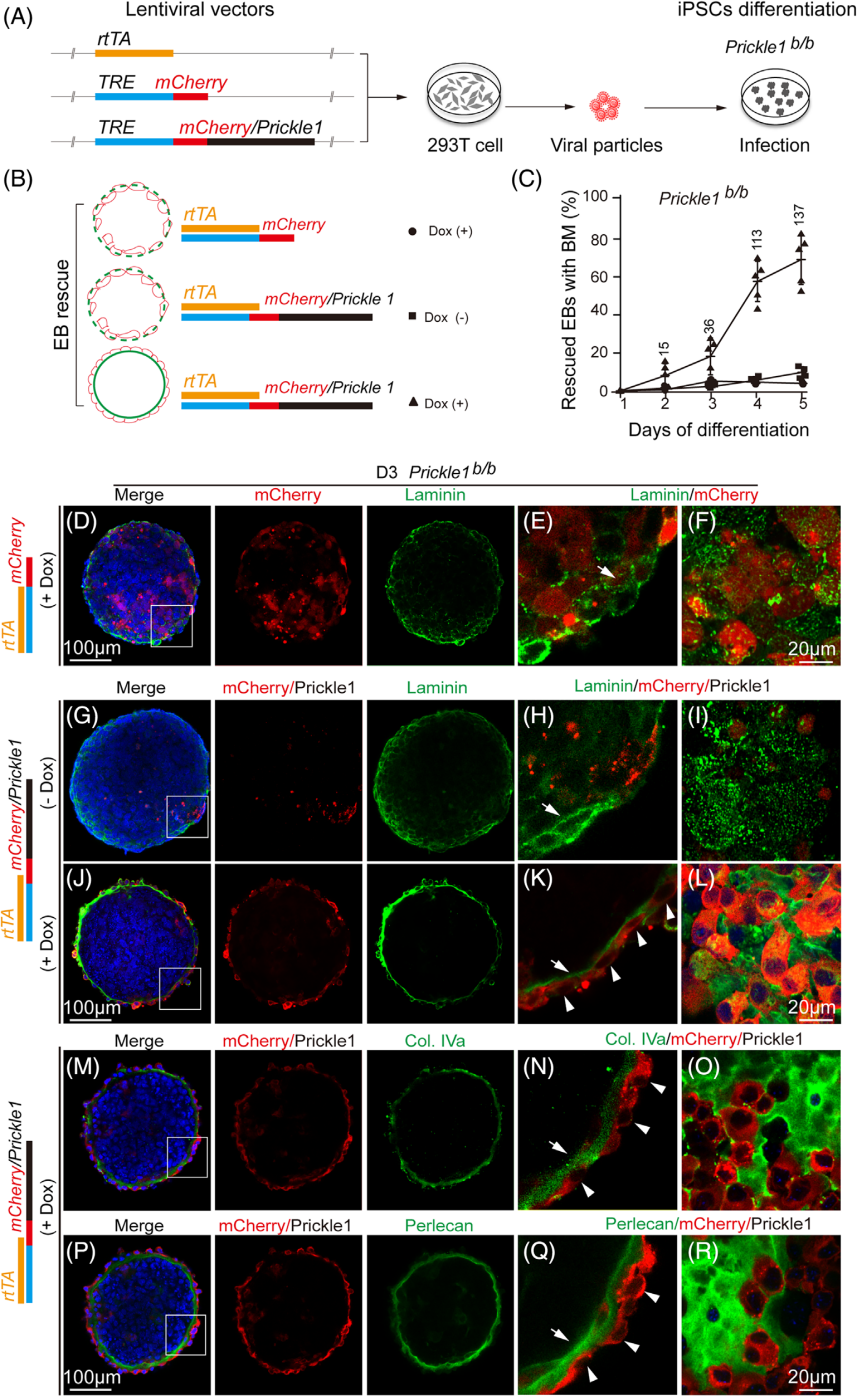
Rescue of BM formation by reintroducing Prickle1 into the mutant EBs. (A) A workflow of constructing lentiviral tetracycline‐inducible vectors, virus production in HEK 293 cells, and virus infection of EBs. By binding to the TRE element, rtTA activates downstream *mCherrry or mCherry/Prickle1* fusion gene expression with the supplement of doxycycline, an analog of tetracycline. (B) Schematic illustration of predicted BM rescue of *Prickle1* mutant EBs. Induced *Cherry*‐carrying mutant EBs with doxycycline (Dox +, solid circle) or uninduced *Cherry/Prickle1*‐carrying mutant EBs (Dox −, solid square) served as controls. Induced *Cherry/Prickle1*‐carrying mutant EBs are indicated by solid triangles. Green lines or dashed lines represent integral or disrupted BM, respectively. (C) BM rescue efficiency is presented as percentages of EBs at different differentiation time points indicated by laminin staining (refer to D–R). Data points of different shapes followed definitions in (B). (D–F) Induced *mCherry* (red) did not rescue BM of the mutant EBs, as indicated by diffused Laminin staining (green). The image in (D) is merged from the mCherry, Laminin, and DAPI channels. The boxed area is magnified in (E) with the arrow points to the laminin staining. (F) An en face view of an immunostained mutant EB with induction of Cherry expression. (G–I) Uninduced *Cherry/Prickle1*‐carrying EBs (Dox −) did not show rescue of basal laminin deposition (green channel). Panel arrangement is the same as in (D–F). (J–L) Induced *Cherry/Prickle1*‐carrying EBs (Dox +) showed rescue of basal laminin deposition (compared to (G–I)). The arrow points to laminin staining. The arrowheads point to the VE cells expressing mCherry/Prickle1. Note the mislocalized Laminin in the VE cells. (M–O) Induced *Cherry/Prickle1*‐carrying EBs (Dox +) showed rescue of basal Col. Iva deposition. The arrow pointing to the BM. The arrowheads point to the VE cells expressing mCherry/Prickle1. (P–R) Induced *Cherry/Prickle1*‐carrying EBs (Dox +) showed rescue of basal Perlecan deposition. The arrow points to the BM. The arrowheads point to the VE cells expressing mCherry/Prickle1.

We next sought to rescue BM and/or cell polarity by adding *Prickle1* back to mutant EBs. Using a lentiviral vector with a built‐in tetracycline‐inducible system, we controlled *Prickle1* expression by adding doxycycline on the day of EB differentiation and harvested at different time points (Figure 6A–C). A *mCherry*‐expressing lentiviral vector served as a control for functional specificity (Figure 6A,B). With the induction of the Prickle1/mCherry fusion protein, the mutant EBs showed continuous BM rescue starting from D2 to D5 of differentiation (Figure 6C, triangles; 6J–R). In contrast, induction of *mCherry* itself (Figure 6C, filled circles; 6D–F) or infection with *mCherry*/*Prickle1* (mCherry‐tagged Prickle1) viruses without doxycycline induction (Figure 6C, squares; 6G–I) showed no significant rescue. The rescued EBs showed basal lamina staining of all three examined BM components, including Laminin (Figure 6J–L), Col IV (Figure 6M–O), and Perlecan (Figure 6P–R). However, the typical columnar shape of the VE cells was not restored by expressing *Prickle1* in the mutant EBs, although many *Prickle1*‐expressing mutant VE cells increased height (Figure 6K,N,Q). While the data suggests that Prickle1 drives BM secretion, it also hints at an imperfection of this rescue system.

### 2.6 AB polarity of the mutant EBs was not rescued by replenishing Prickle1

**FIGURE 7:**
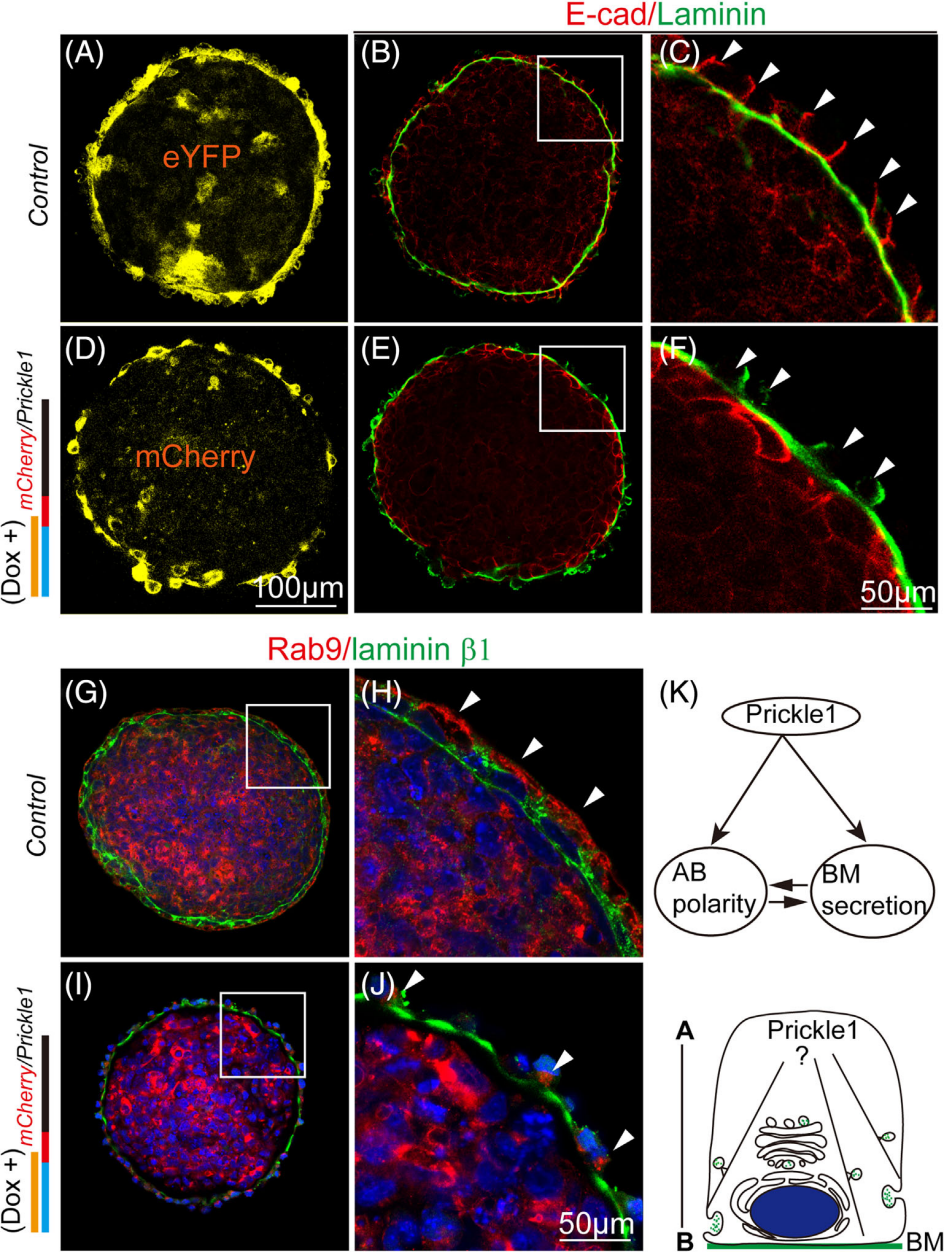
Apical‐basal polarity (AB) polarity is not rescued by reintroducing Prickle1 into the *Prickle1* mutant EBs. (A) eYFP reporter expression from the endogenous *Prickle1* locus of the control EBs as revealed by the anti‐eYFP antibody (yellow). (B) Immunostaining of Laminin (an antibody against all laminin subunits, green) and E‐cadherin (red). The boxed area is magnified next to its right in (C). (C) Magnified boxed area in (B) with arrowheads indicating the basal‐laterally localized E‐cadherin in VE cells. (D) Immunostaining of mCherry to detect the mCherry/Prickle1 fusion protein in the *Prickle1* mutant EBs with Dox induction. (E) Immunostaining of Laminin (green) and E‐cadherin (red). The boxed area is magnified next to its right in (F) with arrowheads indicating the loss of basal‐laterally localized E‐cadherin in VE cells. (G–J) Immunostaining of Rab9 (red) and Laminin β1 (green). DAPI staining is in the blue channel. Boxed areas in (G) and (I) were magnified in (H) and (J), respectively. Arrowheads point to Rab9 localization. Note that Rab9 is apically localized in the control EBs but basally or laterally localized in the ‘Prickle1‐rescue’ EBs. (K) A proposed working model in which Prickle1 regulates AB polarity establishment and basement membrane secretion independently and that AB polarity and BM only interact to form a positive feedback loop once they are established during tissue morphogenesis.

The observation that mutant EBs with the supplement of *Prickle1* still showed defective VE morphology led us to further examine molecular markers for the apical‐basal axis. We first performed immunostaining of E‐cadherin, the most used AB polarity marker for lateral junctions, to determine the re‐establishment of the apicobasal axis. Like the controls (Figure 7A–C), the mutant VE cells expressing Prickle1 expectedly formed BM underlying the VE with some laterally mislocalized laminins (Figure 7D–F). However, basolateral staining of E‐cadherin, as observed in the control VE cells (Figure 7B,C), was not detected in the mutant EBs reexpressing Prickle1 (Figure 7D–F).

Because disruption of vesicle transport prevented directional BM deposition (Supplementary Figure S4), we screened for vesicle trafficking‐related AB polarity markers, particularly focusing on those Rab proteins. Rab proteins are GTPases essential for microtubule‐directed vesicle trafficking in establishing epithelial polarity.^30^ Among the examined Rabs, we found Rab9 had a distinct apical localization pattern in the VE of the control EBs (Figure 7G,H). This localization was essentially reversed in the mutant ‘Prickle1‐rescue’ EBs, with Rab9 localized to the basal‐lateral domain of the VE cells (Figure 7I,J). Thus, the above data suggests that AB polarity is generally not restored in the mutant ‘Prickle1‐rescue’ EBs, and it further implies that BM deposition is relatively separable from the establishment of the AB polarity.

Based on our results, we proposed a working model that Prickle1 regulates AB polarity and BM secretion largely in parallel, and the observations that AB polarity and BM mutually promote (as a positive feedback loop) from other previous studies^6^, ^9^, ^11^, ^12^, ^13^, ^14^ likely only occur after AB and BM are established (Figure 7K).

## 3 DISCUSSION

BM plays an essential role in tissue morphogenesis, providing a physical barrier, drawing tissue boundaries, and coordinating extracellular cell signalling for cell polarization and migration.^5^, ^6^, ^7^, ^8^, ^9^, ^10^ Studies in chordates,^10^ flies,^9^, ^11^ mice,^23^, ^24^ and MDCK cells^12^ suggest crosstalk between BM and cell polarity involving vesicle transport,^6^, ^15^ lipid regionalization,^14^ and cytoskeleton reorganization.^13^ Particularly, Prickle1, a component of the planar cell polarity module (Frizzled/Dishevelled/Vangl/Prickle/Diego/and Flamingo), is involved in both apicobasal polarity and BM deposition crucial for embryogenesis and tissue morphogenesis.^10^, ^21^, ^23^, ^24^, ^31^, ^32^, ^33^, ^34^, ^35^, ^36^, ^37^ It is generally assumed that BM basal deposition is a result of apicobasal polarity. However, the coupled BM and cell polarity phenotypes of *Prickle1* mutants make it challenging to dissect which one is the causal or consequential event.

The current study attempts to tackle the above problem using in vitro differentiated EBs, which possess BM, separating the visceral endoderm and the inner EB cell mass. EBs were derived from *Prickle1* mutant and normal embryonic fibroblast cells through iPSC differentiation. Similar to that observed in the embryo or tear duct development,^23^, ^24^ mutant EBs with disruption of *Prickle1* do not develop integral BM and have impaired cell polarity. The BM secretion appeared to be VE‐dependent based on two observations: (i) *Prickle1* is highly expressed in VE cells of the EBs, and *Prickle1* mutant VE do not form BM, and (ii) mutant VE cells displayed abnormally flattened morphology compared with the normal columnar shape of the controls. However, these observations do not discriminate whether the BM defect is primary or secondary to the cell polarity defect. Reintroduction of *Prickle1* into the mutant EBs rescues BM deposition but not the AB polarity (though some mutant cells replenished with Prickle1 partially reversed the flat morphology) suggests that the mutant BM defect is indeed independent of AB polarity.

One caveat for the above interpretations is that the mutant VE has fewer cells, which might cause insufficient ECM secretion, compromising the BM assembly. However, this appears to be not the case since Prickle1‐rescue EBs have a similar lower number of VE cells but with a completely rescued BM. Thus, the regulation of BM deposition by Prickle1 is also less dependent on the VE cell number. The fewer VE cells of the mutant EBs likely reflect other functional aspects of Prickle1 (for example, vectorial cell migration), which requires further investigation.

The regulation of Prickle1 on BM secretion probably involves microtubule‐directed vesicle secretion. Loss of Prickle1 altered cytoskeleton and vesicle compartments of the epiblast and the embryonic tear‐duct epithelial cells.^23^, ^24^ Interestingly, it is the disruption of microtubules or vesicle trafficking but not actin assembly that completely abolished BM. In an effort to search for proteins responsible for microtubule‐dependent BM vesicle trafficking, we focused on Rab small GTPases and found that Rab9 is apically polarized in VE, and its localization is randomized in the mutant EBs. However, Rab9 localization is not essential for BM deposition since BM is rescued without restoration of Rab9 polarization, another indicator of AB polarity. How Prickle1 directs microtubule network and vesicle trafficking requires future detailed characterization.

The reasons for the failure in rescuing the AB polarity of the *Prickle1* mutant EBs are probably complicated. The VE cell number and the variable timing and dosage of replenished *Prickle1* expression would all contribute to AB restoration. VE cell number might affect lateral junction formation, whilst either over‐ or under‐ expression of cell polarity components (in this case, Prickle1) would lead to the same orientation defects. Further refined tools are needed to address these issues.

Although the initial BM formation is largely independent of AB polarity, as revealed by this study, studies from other systems suggest that basal BM is required for the AB polarity,^9^, ^10^, ^11^, ^12^ and AB polarity is also crucial for BM basal deposition.^24^ The seemingly paradox observations could be explained when considering that AB polarity and BM basal deposition form a positive feedback loop only after their establishment (Figure 7K), which is another interesting question worthy of future investigation.

Another implication from this study is that VE might play a determinant role in BM secretion at the junction of VE and epiblast before gastrulation. This is evidenced by the high expression of *Prickle1* in the VE cells of the EBs and the role of Prickle1 in dictating BM secretion in multiple tissues.^21^, ^23^, ^24^ Recently, we also found that *Prickle1* expression in the VE is much stronger than the epiblast in early embryogenesis. Thus, a careful in vivo characterization of the BM defect using *Prickle1* conditional knockout mice combined with VE or epiblast specific Cre lines will be needed in the future to clarify which germ layer dominates BM secretion.

## 4 MATERIALS AND METHODS

### 4.1 Mice and genotyping

Animal husbandry and experimentation were conducted in strict adherence to the Standards in Animal Research: Reporting of In Vivo Experiments (ARRIVE) guidelines, with approval from the Animal Care and Use Committee (ACUC), Zhongshan Ophthalmic Center, Sun Yat‐sen University. Mouse strains were of mixed genetic backgrounds of C57BL/6 and Sv129. *Prickle1* gene‐trap mutant strain was created as described previously.^21^, ^32^ *Prickle1* null allele (*Prickle1* ^*b/+*^) was created by excision of Cre recombinase driven by *Sox2* promoter (So*x2‐Cre*).^21^ Mouse genotyping was conducted as described previously.^21^, ^32^ A knock‐in *eYFP* reporter under the control of endogenous *Prickle1* promoter was used to monitor *Prickle1* expression.

### 4.2 Generation and characterization of iPSCs from MEFs

Isolation, culturing, and maintenance of mouse embryonic fibroblasts (MEFs) followed standard protocols.^27^ Briefly, wild type, *Prickle1* heterozygous (*Prickle1* ^*b/+*^ *)* and mutant embryos (*Prickle1* ^*b/b*^ *)* were collected at post‐coitus day E13.5. After removing the head and viscera, the remaining trunk was subjected to trypsin digestion and cultured in DMEM.

For iPS cells (iPSCs) induction, MEFs were reprogrammed into iPSCs by lentiviral particles expressing ‘4 factors’ under the control of a tet‐inducible expression system.^28^ Briefly, TetO‐FUW‐OSKM and FUW‐M2rtTA (Addgene) lentiviral vectors were separately transfected into HEK 293 cells together with pMD2.G, pMDLg/pRRE and pRSV‐Rev helper virus vectors (Addgene). The culture medium was collected and ultracentrifuged to concentrate viral particles. Cultured MEFs were then infected with the packaged OSKM and rtTA viruses separately, with doxycycline (SD8430, Solarbio, China) induction. iPSCs can be observed 5 days after infection. All plasmid information can be found at: https://www.addgene.org/20321/ (for TetO‐FUW‐OSKM), https://www.addgene.org/20342/ (for FUW‐M2rtTA), https://www.addgene.org/12259/ (for pMD2.G), https://www.addgene.org/12251/ (for pMDLg/pRRE), and https://www.addgene.org/12253/(for pRSV‐Rev).

The derived‐iPSCs were first characterized by alkaline phosphatase staining according to the manufacturer's instructions (MA0197, Meilunbio, China), then subjected to immunohistochemistry for examination of a set of stem cell markers including Sox2, Nanog, and SSEA1. Teratoma formation was used to functionally evaluate the pluripotency of iPSCs by subcutaneous injection of 1 × 10^6^ cells into immunodeficient BALB/c‐Nude mice (BALB/cNj‐Foxn1nu/Gpt, GemPharmatech, strain NO. D000521). Mice were sacrificed at 2.5 months after injection, followed by H&E staining to identify tissue types.

### 4.3 Karyotyping analysis

Two iPSC lines (each for *Prickle1* ^*b/+*^ *and Prickle1* ^*b/b*^ clones) were subjected to karyotyping analysis. The mouse iPSC cells were cultured in MEF feeder layers to 70%–80% density and treated with 0.5 μM colcemid (234,115, Sigma) in iPSC growing medium for 2 hours before harvesting. After digested with 0.25% trypsin–EDTA, the iPSCs were collected and centrifuged at 200*g* for 5 min. The supernatant was removed, and cell pellets were resuspended with 10 mL 75 mM KCl solution and incubated for 20 min at 37°C. Then the cells were centrifuged for 10 min at 500*g* and fixed in 10 mL fixative solution (methanol/acetic acid 3:1) twice, with 10 min centrifugation at 500*g* in between. Finally, the cells was resuspended in cold methanol/acetic acid fixative solution and spread onto glass slides for dry, 3 h at 75°C. The slides were then treated with 0.0025% trypsin for 5 min and stained with Giemsa (10% in PBS) prepared for karyotype imaging analysis with Ikaros karyotyping system (Imager‐Z2, ZEISS).

### 4.4 Viral integration and expression analysis

To determine whether the used iPSC clones are devoid of exogenous ‘OSKM’ expression, we designed specific PCR primers for T2A and E2A elements flanking *Klf4* coding sequence of the ‘OSKM’ vector (Supplementary Figure S2C). ‘OSKM’ expression and viral vector integration were detected by RT‐qPCR and PCR analysis using reversely transcribed cDNA and genomic DNA from iPSCs, respectively. The iPSCs were treated with doxycycline at 1 μg/mL for 3 days to induce potential ‘OSKM’ expression, meanwhile, a MEF line with expressible integrated‐ ‘OSKM’ viral vector and an ES line with no viral vector infection serve as positive and negative controls, respectively. T2A forward primer: 5′‐CGGGGACGTGGAGGAAAATC‐3′; E2A reverse primer: 5′‐TGCTCTCAACATCTCCAGCC‐3′.

### 4.5 EB differentiation and rescue of the mutant EBs

The derived iPSCs were differentiated into embryoid bodies (EBs) according to the protocol described previously.^26^ Briefly, iPSCs were grown on MEF cells (treated with Mitomycin C) in DMEM culture medium (10569044, Thermo Fisher) with 15% FBS (SH30071, Cytiva), 1% NEAA (11140050, Thermo Fisher), 1% Penicillin/Streptomycin (15140122, Thermo Fisher), 0.1 mM β‐mercaptoethanol (21985023, Thermo Fisher), and 1000 U/mL leukaemia inhibitory factor (ESG1106, Millipore). For EB differentiation, 1X10^3^ single iPSCs were seeded in one well of a 6‐well plate and cultured for 4 days until reaching a desirable size of about 200 μm for the ensuing aggregation. Clones were lifted with 0.25% Trypsin–EDTA (25200072, Thermo Fisher) and naturally aggregated into clumps (~100 μm), which were plated onto gelatin‐coated plates for 2 h, allowing residual feeder cells to attach. The iPSC aggregates were then transferred onto bacteriological petri dishes with iPSC culture medium (without LIF) and cultured for 3 days.

For the rescue experiment, mCherry‐tagged Prickle1 (mCherry/Prickle1) and mCherry cDNAs were separately cloned into a lentiviral vector by replacing the OSKM cassette of the TetO‐FUW‐OSKM vector. Virus packaging was performed by transfecting each construct into HEK 293 together with helper virus vectors, as described in the previous section. *Prickle1* ^*b/b*^ mutant EBs were infected with mCherry/Prickle1 and mCherry viruses at differentiation day 0 (D0) with the addition of doxycycline. Successfully differentiated EBs can be identified with an outer layer of visceral endoderm having *Prickle1* expression (indicated by a knock‐in EYFP reporter).

### 4.6 Immunohistochemistry, Western blot, and antibodies

For immunostaining, EBs were fixed with 4% PFA for 10 min, washed with PBS three times, and then subject to immunostaining according to the standard protocol. Primary antibodies used in this study are: Anti‐E‐cad (ab11512, Abcam), anti‐eYFP/GFP (TP401, Torrey pines Biolabs), anti‐eYFP/GFP (ab6673, Abcam), anti‐Laminin (L9393, Sigma), anti‐Lamininβ1 (ab44941, Abcam), anti‐Collagen IV (ab19808, Abcam), anti‐Perlecan (MA1‐06821, Invitrogen), anti‐Ac‐Tubulin (T6793, Sigma), anti‐Nanog (ab80892, Abcam), Anti‐Sox2 (sc365964, Santa Cruz), anti‐Integrinβ1 (ab179471), anti‐Gapdh (NBP2‐27103, NOVUS), anti‐Rab6a (bs‐11259R, Bioss), anti‐Rab8 (55296‐1‐ap, Proteintech), anti‐Rab9 (ab179815, Abcam), anti‐Rab10 (ab237703, Abcam), anti‐Rab11 (ab95375, Abcam), and Anti‐Ssea1(ab16285, Abcam), and Tra‐1‐60 (ab16288，Abcam), Oct4 (ab19857, Abcam), and anti‐c‐Myc (cst5605, Cell Signalling Technology). Actin fibres were stained with 488‐ Phalloidin (A12379, Thermo Fisher), 568‐ Phalloidin (A12380, Thermo Fisher).

The secondary antibodies used for staining are: Alexa‐Fluor568 Donkey anti‐rabbit IgG (H + L) (A10042, Thermo Fisher), Alexa‐Fluor488 Donkey anti‐rabbit IgG (H + L) (A‐21206, Thermo Fisher), Alexa‐Fluor488 Donkey anti‐mouse IgG (H + L) (A‐21202, Thermo Fisher), and Alexa‐Fluor568 Donkey anti‐Rat IgG (H + L) (ab175475, Abcam).

For western blotting, proteins from both control and *Prickle1* mutant EBs at D3 were extracted with RIPA buffer (R0278; Sigma‐Aldrich) with a protease inhibitor cocktail (P8340; Sigma‐Aldrich). After centrifugation, the supernatant was collected and prepared for Western blotting according to a standard protocol as previously described.^38^ All primary antibodies for Western blotting (see the above antibody list for immunohistochemistry) were diluted at 1:1000–1: 2000 dilution. Secondary HRP‐conjugated antibodies, anti‐Rabbit IgG (ab6721, Abcam) and anti‐Rat IgG (ab97057, Abcam), were used at 1:5000 dilution.

### 4.7 Drug treatment

EBs at differentiation D3 were treated with Cytochalasin D (final concentration 10 μM, PHZ1063, Thermo Fisher) and Nocodazle (final concentration 0.5 μg/mL, M1404‐2MG, Sigma). Vehicle (DMSO) only was used in control groups. EBs were harvested at 2 and 10 h posttreatment, washed with PBS several times, and fixed with PFA for further immunohistochemistry and imaging analysis.

### 4.8 RNA isolation and RT‐qPCR

For measuring gene expression, three biological replicates of both controls and *Prickle1* mutant EBs were subjected to qPCR analysis. Total RNA was extracted using the Trizol reagent kit (15596026, Invitrogen) according to the manufacturer's protocol. The RNAs were reverse transcribed into cDNAs with HiScript III All‐in‐one RT SuperMix Perfect for qPCR (R333; Vazyme). Real‐time PCR was performed by using iTaq™ Universal SYBR® Green Supermix kits (1725120, Bio‐Rad) according to the manufacturer's instructions. Gene expression (Ct values) was normalized to the GAPDH. Graphs were plotted using GraphPad Prism 9 software. Statistics was performed by Student's *t*‐test, and *p* < 0.05 is designated to be significant. Primers used for RT‐PCR are listed in Supplementary Table S1.

### 4.9 Counting and statistical analysis

For EB differentiation, EBs with Gata4‐positive staining on the outer layer were designated as successful differentiation. The total of EBs from three independent experiments with or without surficial Gata4 staining of the EBs were subjected to statistical analysis using chi‐square distribution.

To evaluate the EB defects upon exposures to cytochalasin D or nocodazole, we categorized the EBs into three groups: EBs with integral BM and no cytoplasmic accumulation of laminin were designated as ‘normal’ (Figure 5M, blue icon); EBs with compromised BM structure and cytoplasmic laminin accumulation were designated as ‘compromised’ (Figure 5M, green icon); and EBs with on apparent BM structure were designated as ‘disrupted’ (Figure 5M, red icon). The total of EBs stained for laminin from three independent experiments were subjected to statistical analysis using chi‐square distribution according to the above set criteria. *p* < 0.05 is designated to be significant.

### 4.10 Imaging analysis

All fluorescence microscopy images were obtained using Zeiss confocal microscope (Zeiss LSM880, Zeiss, Germany). Different imaging channels were given distinct pseudo colours.

## AUTHOR CONTRIBUTIONS

Dianlei Guo conducted EB differentiation and drafted the manuscript. Sikai Liu performed immunostaining. Jiao Zhang prepared tissue sections. Xinyu Gu performed viral packaging. Lei Shi assisted with MEFs, iPS reprogramming, and karyotyping. Yingchun Su prepared viral expression vectors. Rong Ju performed EB drug treatment. Yanhong Wei analysed data and provided intellectual discussion. Chunqiao Liu conceived the study, interpreted the data, and wrote final version of the paper.

## FUNDING INFORMATION

This work is supported by National Science Foundation Youth Program (32000553) to Dianlei Guo, Guangdong Provincial Natural Science Foundation (2022A1515012515), Guangzhou City‐University Joint Foundation (202201020275), and Research funding from State Key Laboratory of Ophthalmology at Zhongshan Ophthalmic Center (303060202400373) to Chunqiao Liu.

## CONFLICT OF INTEREST STATEMENT

We declare no conflicts of interest associated with this work.

## Supporting information


**Data S1:** Supporting Information.

## Data Availability

All data supporting the current study can be obtained through communicating with the corresponding authors.
