# Supplementary material for: Prickle1‐driven basement membrane deposition of the iPSC‐derived embryoid bodies is separable from the establishment of apicobasal polarity
Source: Cell Prolif. 2024 Jan 7;57(6):e13595. doi: 10.1111/cpr.13595 (PMC11150132; doi:10.1111/cpr.13595)
Supplement: Supplementary file 1 — Data S1: Supporting Information. [file CPR-57-e13595-s001.pdf]

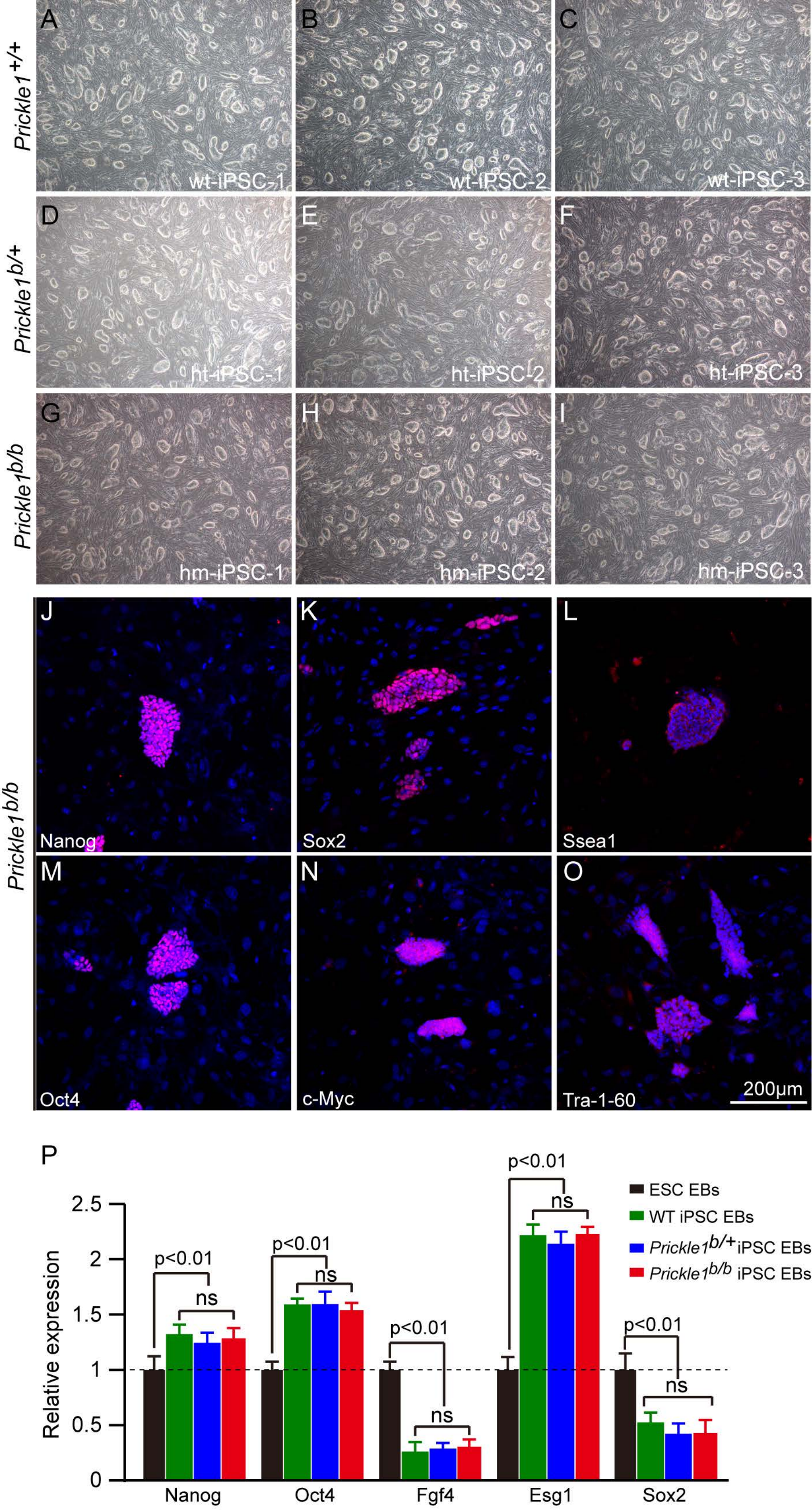

Supplementary Figure 1

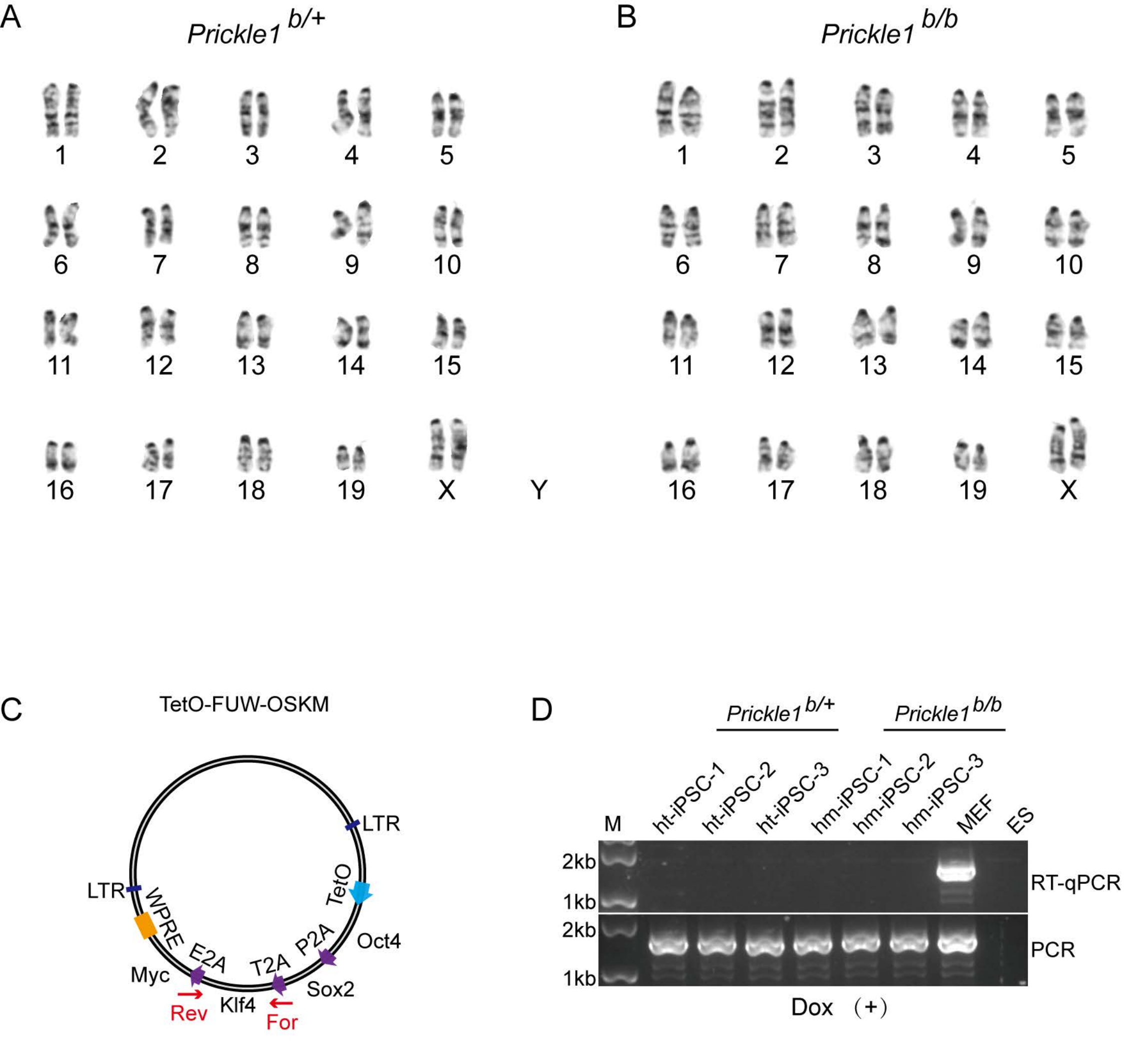

Supplementary Figure 2

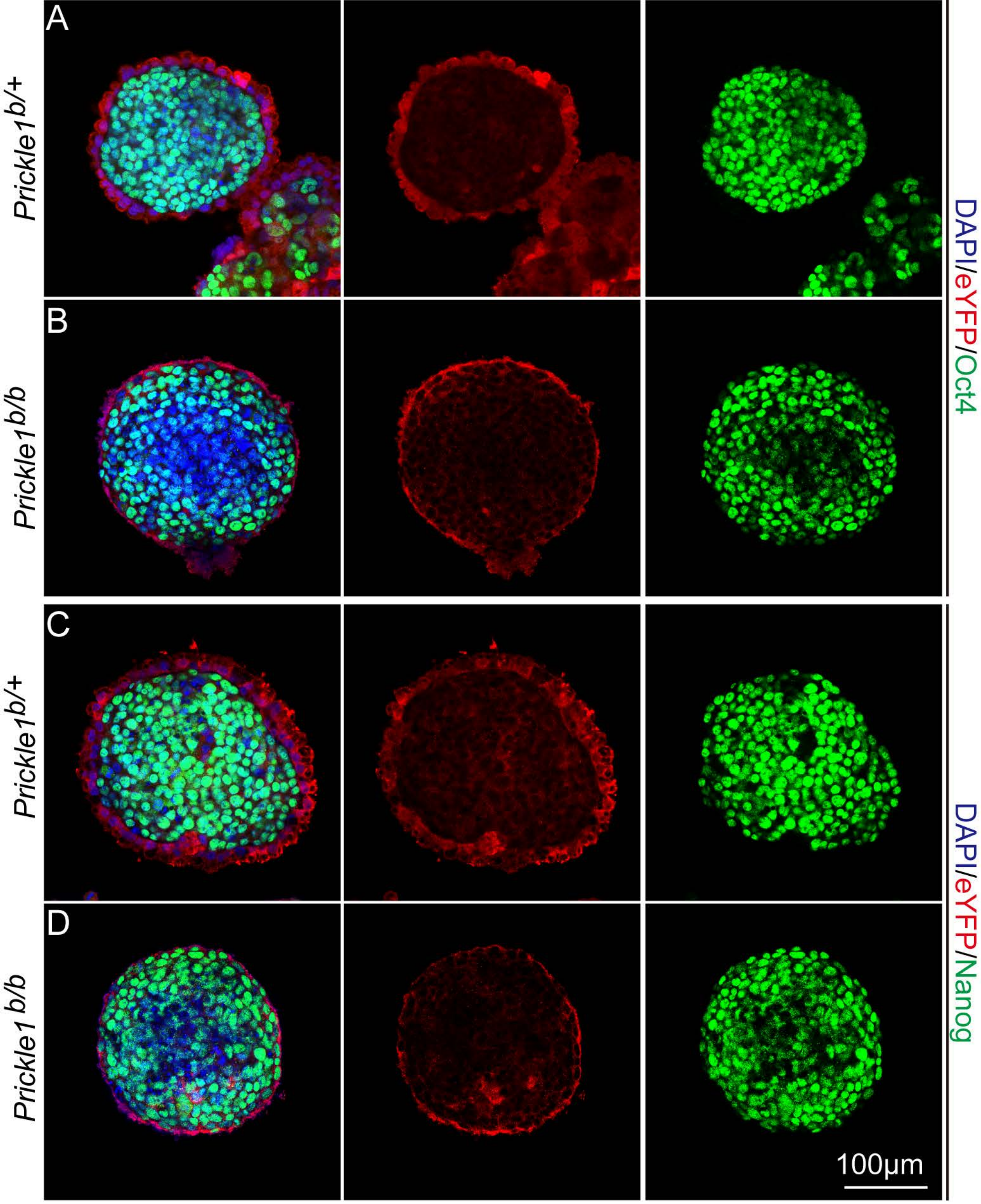

Supplementary Figure 3

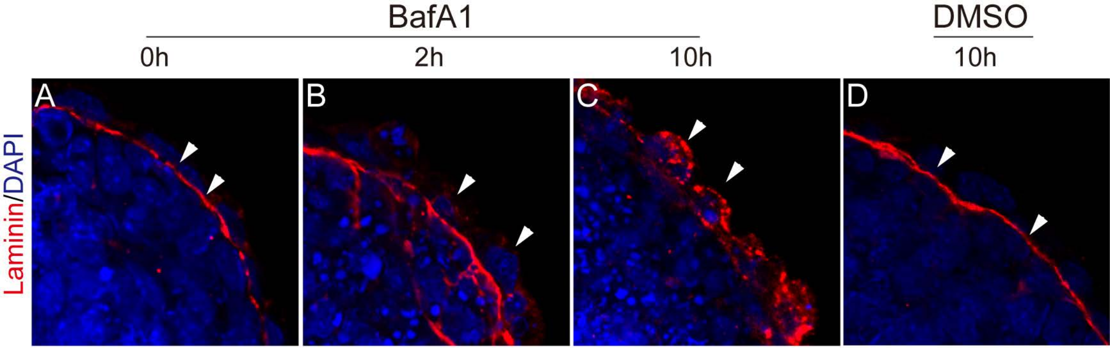

Supplementary Figure 4

Supplementary Figure 1. Validation of iPSCs. (**A-I**), Morphology of reprogrammed iPSC clones. Three representative iPSC lines are shown for each genotype: (**A-C**) *Prickle1*$^{+/+}$ (wt-iPSC-1, wt-iPSC-2, and wt-iPSC-3); (**D-F**) *Prickle1*$^{b/+}$ (ht-iPSC-1, ht-iPSC-2, and ht-iPSC-3); (**G-I**) *Prickle*$^{b/b}$ (hm-iPSC-1, hm-iPSC-2, and hm-iPSC-3). (**J-O**), iPSC clones from *Prickle*$^{b/b}$ immunostained for Nanog (**J**), Sox2 (**K**), SSEA1 (**L**), Oct4 (**M**), c-Myc(**N**), and Tra-1-60 (**O**). (**P**), RT-qPCR to determine the mRNA expression of genes encoding for iPSC key markers. RT-qPCR CT values of each gene were normalized to the *Gapdh* followed by calculation of relative ratios to that of ESCs. Student t-test was performed to detect statistical powers p-values. Statistical significance was defined as $p<0.05$.

Supplementary Figure 2. Karyotyping analysis and detection of the "OSKM" viral vector expression and integration. (A), Chromosome pairs of a "OSKM"-induced *Prickle1* $^{b/+}$ iPSC line. **(B),** Chromosome pairs of a "OSKM"-induced *Prickle1* $^{b/b}$ iPSC line. **(C),** Illustration of the "OSKM" viral vector. The primers used for detecting expression and integration were indicated with red arrows (Materials and Methods). **(D),** The three *Prickle1* $^{b/+}$ and *Prickle1* $^{b/b}$ iPSC clones in Supplementary Figure 1 were examined for "OSKM" transcript expression and genome integration. All iPSC clones were Dox-induced for 3 days before performing RT-qPCR and PCR analysis. An expressible "OSKM" -integrated MEF line was used as a positive control,

whereas an ES line without viral vector infection served as a negative control.

Supplementary Figure 3. Immunohistochemistry to detect Oct4 and Nanog expression in the inner EB cell mass. (**A, B**), DAPI (blue), Oct4 (Green), and eYFP (Red). (**A**), Control EBs. (**B**) *Prickle*$^{b/b}$ mutant EBs. (**C, D**) Nanog (Green). (**C**) Control EBs. (**D**) *Prickle*$^{b/b}$ mutant EBs.

Supplementary Figure 4. Treatment of control EBs with bafilomycin A1 (BafA1) led to disruption of the BM. (**A-D**), BM integrity after BafA1 treatment for 0hr (**A**), 2hr (**B**), and 10hr (**C**). (**D**) DMSO treatment as a sham control. Red, Laminin staining; Blue, DAPI staining.
